# Evaluation of Claims-Based Ascertainment of Alzheimer Disease and Related Dementias Across Health Care Settings

**DOI:** 10.1001/jamahealthforum.2022.0653

**Published:** 2022-04-22

**Authors:** Natalia Festa, Mary Price, Lidia M. V. R. Moura, Deborah Blacker, Sharon-Lise Normand, Joseph P. Newhouse, John Hsu

**Affiliations:** 1 VA Office of Academic Affiliations through the VA/National Clinician Scholars Program and Yale University, New Haven, Connecticut; 2 Mongan Institute, Massachusetts General Hospital, Harvard Medical School, Boston; 3 Department of Neurology, Massachusetts General Hospital, Boston; 4 Department of Psychiatry, Massachusetts General Hospital, Boston; 5 Department of Health Care Policy, Harvard Medical School, Boston, Massachusetts; 6 Harvard University, Cambridge, Massachusetts

## Abstract

This cohort study evaluates the ascertainment of Alzheimer disease and related dementia using diagnostic codes in various health care settings.

## Introduction

The accuracy with which claims-based diagnostic codes capture Alzheimer disease and related dementias (ADRD) may vary across health care settings,^1^ thereby affecting the validity of research, risk adjustment, or population health management strategies predicated on these measures. Ascertainment of ADRD using diagnostic codes from hospital encounters may be biased by fragmented access to relevant clinical information, the presence of acute illnesses that alter or preclude clinical assessments,^2,3,4^ and the inclusion of problematic diagnostic codes within common claims-based definitions of ADRD. Delirium, a potential neuropsychiatric complication of hospitalization,^5^ may be conflated with dementia and is included in the common US Centers for Medicare & Medicaid Services Chronic Conditions Warehouse (CCW) claims-based definition of ADRD. To illustrate, we described the performance of the CCW ADRD definition in discriminating clinician-adjudicated disease status across health care settings.

## Methods

We used longitudinal, individual-level data from the Mass General Brigham accountable care organization to construct a cohort of 37 200 Medicare beneficiaries whom we observed from January 1, 2016, to December 31, 2018,^6^ as approved by the Mass General Brigham institutional review board. Informed consent was waived for this population based on the observational nature of the study and the use of historical data. Additional methodological details of the inclusion criteria, sample, and reference standard construction are available in a prior publication.^6^ We followed the Strengthening the Reporting of Observational Studies in Epidemiology(STROBE) reporting guideline.

Three expert clinicians reviewed electronic health record data to adjudicate ADRD status. We compared the clinician-adjudicated classification of beneficiaries with their qualifying claims-based dementia diagnoses within the CCW ADRD definition. We classified beneficiaries who met CCW criteria but did not have ADRD on the review of the electronic record as *false-positives* and those with clinician-adjudicated ADRD as *true-positives*.

We described the characteristics of true-positives and false-positives based on the health care setting in which their qualifying CCW ADRD diagnostic codes were documented: (1) hospital-only claims; (2) outpatient-only claims; or (3) claims from 2 or more distinct health care settings (hospital, emergency department, outpatient, skilled nursing facility, home health carrier, and hospice). We estimated 3 logistic models in which we regressed clinician-adjudicated dementia on the CCW classification of ADRD for each health care setting subgroup. We reported the prevalence of true-positives and false-positives as well as the mean and 95% CI for the area under the receiver operating characteristic curve (AUROC). We summarized the prevalence of the most frequently assigned diagnostic claims within the CCW ADRD definition by setting. Analyses were conducted using Stata, version 17 (StataCorp).

## Results

Table 1 displays the characteristics of the 3873 beneficiaries with claims-based diagnoses who met the CCW ADRD definition. Among beneficiaries with hospital-only ADRD diagnoses (566 [14.6%]), 391 (69%) were false-positives compared with 455 beneficiaries (52%) with outpatient-only diagnoses and 490 beneficiaries (23%) with diagnoses from at least 2 distinct health care settings (Table 2). The accuracy of the CCW definition was poorest among beneficiaries with hospital-only ADRD diagnoses (AUROC, 0.51; 95% CI, 0.49-0.52). Accuracy improved somewhat for those with outpatient-only ADRD diagnoses (AUROC, 0.57; 95% CI, 0.54-0.60) and substantially for diagnoses from multiple distinct health care settings (AUROC, 0.81; 95% CI, 0.78-0.84). The most common diagnostic codes among false-positives were either noncognitive (eg, age-related physical debility) or corresponded to distinct neuropsychiatric syndromes (eg, delirium) across each health care setting.

**Table 1.** Characteristics and Health Care Utilization of Beneficiaries With Qualifying CCW ADRD Claims by Health Care Setting

Characteristic	No. %						
	Clinician-adjudicated dementia	ADRD claims, only				ADRD claims from ≥2 health care settings	
		Hospital		Outpatient			
		FP	TP	FP	TP	FP	TP
**Beneficiaries, No.**	**2806**	**566**	**255**	**455**	**421**	**490**	**1686**
Age, mean (SD), y	83 (0.7)	81 (1.5)	80 (3.1)	80 (1.8)	78 (1.3)	82 (1.2)	84 (0.6)
Sex							
Female	1796 (64)	317 (56)	112 (44)	296 (65)	290 (69)	319 (65)	1130 (67)
Male	1010 (36)	249 (44)	143 (56)	159 (35)	131 (31)	171 (35)	556 (33)
Cancer	334 (12)	143 (25)	65 (26)	69 (15)	87 (21)	113 (23)	182 (11)
Diabetes	360 (13)	151 (27)	69 (27)	87 (19)	35 (8)	139 (28)	226 (13)
CHF	449 (16)	103 (18)	134 (52)	35 (8)	9 (2)	182 (37)	191 (11)
COPD	243 (9)	87 (15)	0	9 (2)	26 (6)	82 (17)	187 (11)
Stroke	104 (4)	0	17 (7)	9 (2)	9 (2)	17 (4)	78 (5)
Resident of low SES zip code^a^	186 (7)	26 (5)	0	26 (6)	0	17 (4)	69 (4)
No. of hospital admissions, mean (SD), y	0.6 (0.1)	1.0 (0.2)	1.0 (0.2)	0.2 (0.1)	0.1 (0.1)	0.6 (0.1)	0.7 (0.1)
Hospital admissions, mean (SD), d/y	3.9 (0.5)	7.4 (1.6)	5.2 (0.7)	1.1 (0.4)	0.3 (0.1)	3.7 (0.8)	4.9 (0.8)
Institutional postacute care, mean (SD), d/y	5.7 (0.7)	10.0 (2.3)	7.3 (3.9)	0.9 (0.4)	0.3 (0.2)	8.2 (1.8)	6.9 (0.8)
Home health utilization, mean (SD), d/y	25.7 (3.1)	12.5 (4.1)	32.9 (11.8)	5.8 (2.1)	4.2 (2.4)	22.7 (4.3)	30.0 (3.8)
No. of primary care visits, means (SD), y	3.4 (0.2)	4.5 (0.5)	3.5 (1.0)	3.1 (0.6)	3.1 (0.4)	4.5 (0.4)	3.4 (0.3)

Abbreviations: ADRD, Alzheimer disease and related dementias; CCW, US Centers for Medicare & Medicaid Services Chronic Conditions Warehouse; CHF, congestive heart failure; COPD, chronic obstructive pulmonary disease; FP, false-positive; SES, socioeconomic status; TP, true-positive.

^a^ Low SES zip code if more than 25% of residents 25 years or older with less than a high school diploma or more than 20% of residents with an income less than 100% of poverty line based on the 2015 to 2019 American Community Survey.

**Table 2.** Overlap of CCW ADRD Definition With Clinician-Adjudicated Dementia by Health Care Setting

Beneficiaries with qualifying diagnostic claim	No. (%)		Total
	Clinician-adjudicated dementia		
	Yes	No	
Total patients	2806	34 394	37 200
**ADRD claims**			
Hospital only			
Yes	255 (31)	566 (69)	821
No	2551 (7)	33 827 (93)	36 378
Outpatient only			
Yes	421 (48)	455 (52)	876
No	2385 (7)	33 939 (93)	36 324
From ≥2 health care settings			
Yes	1686 (77)	490 (23)	2176
No	1120 (3)	33 904 (97)	35 024

Abbreviations: ADRD, Alzheimer disease and related dementias; CCW, US Centers for Medicare & Medicaid Services Chronic Conditions Warehouse.

## Discussion

Greater than two-thirds of beneficiaries whose CCW ADRD diagnoses were present only within hospital claims were classified as false-positives by the reference standard. When operationalized using hospital-only diagnoses, the CCW ADRD definition performed no better than chance in discriminating clinician-adjudicated dementia status. This setting-dependent decrement in diagnostic accuracy is consistent with the known challenges of discerning ADRD during hospital encounters.^4^ Caution is warranted in interpreting research findings, risk adjustment, or population health management interventions predicated on hospital-only ADRD diagnoses. The clinician-adjudicated reference standard is limited to information available within the electronic health record, data that are contingent on evaluation and documentation by clinicians. Incorporating metadata regarding the setting or frequency of diagnostic claims could improve the accuracy with which ADRD is ascertained for research, population health, or reimbursement purposes.
